# Cadaveric analysis of transcranial versus endoscopic transorbital petrosectomy: comparison of surgical maneuverability and brainstem exposure

**DOI:** 10.3389/fonc.2023.1186012

**Published:** 2023-07-06

**Authors:** Sae Min Kwon, Min Kyun Na, Kyu-Sun Choi, Hyoung Soo Byoun, Yong Seok Nam

**Affiliations:** ^1^ Department of Neurosurgery, Dongsan Medical Center, Keimyung University School of Medicine, Daegu, Republic of Korea; ^2^ Department of Neurosurgery, College of Medicine, Hanyang University, Seoul, Republic of Korea; ^3^ Department of Neurosurgery, Chungnam National University Sejong Hospital, Sejong, Republic of Korea; ^4^ Department of Anatomy, College of Korean Medicine, Dongshin University, Naju, Republic of Korea

**Keywords:** transorbital approach, endoscope, brainstem, anterior petrosectomy, skull base

## Abstract

**Introduction:**

While accessing the posterior fossa, the anterior transpetrosal approach (ATPA) and endoscopic transorbital approach (ETOA) use the same bony landmarks during petrous apex drilling. However, owing to their contrasting surgical axes, they are expected to show differences in surgical view, maneuverability, and clinical implications. This study aimed to investigate the feasibility of ETOA in accessing the brainstem and to compare the surgical view and maneuverability of each approach.

**Methods:**

ATPA and ETOA were performed in four human cadaveric heads (eight sides and four sides in each procedure). The angle of attack (AOA) and surgical depth were measured at the target of interest (root exit zone [REZ] of cranial nerve [CN] V, VI, and VII). When measuring the area of exposure, the brainstem was divided into two areas (anterior and lateral brainstem) based on the longitudinal line crossing the entry zone of the trigeminal root, and the area of each was measured.

**Results:**

ATPA showed significantly greater value at the trigeminal REZ in both vertical (31.8 ± 6.7° vs. 14.3 ± 5.3°, p=0.006) and horizontal AOA (48.5 ± 2.9° vs. 15.0 ± 5.2°, p<0.001) than ETOA. The AOA at facial REZ was also greater in ATPA than ETOA (vertical, 27.5 ± 3.9° vs. 8.3 ± 3.3°, p<0.001; horizontal, 33.8 ± 2.2° vs. 11.8 ± 2.9°, p<0.001). ATPA presented significantly shorter surgical depth (CN V, 5.8 ± 0.5 cm vs. 9.0 ± 0.8, p<0.001; CN VII, 6.3 ± 0.5 cm vs. 9.5 ± 1.0, p=0.001) than ETOA. The mean area of brainstem exposure did not differ between the two approaches. However, ATPA showed significantly better exposure of anterior brainstem than ETOA (240.7 ± 9.6 mm^2^ vs. 171.7 ± 15.0 mm^2^, p<0.001), while ETOA demonstrated better lateral brainstem exposure (174.2 ± 29.1 mm^2^ vs. 231.1 ± 13.6 mm^2^, p=0.022).

**Conclusions:**

ETOA could be a valid surgical option, in selected cases, that provides a direct ventral route to the brainstem. Compared with ATPA, ETOA showed less surgical maneuverability, AOA and longer surgical depth; however, it presented comparable brainstem exposure and better exposure of the lateral brainstem.

## 1 Introduction

Surgical access to the petrous apex and petroclival area remains challenging owing to the anatomical complexity, deep location, and proximity to critical neurovascular structures (1–3). The anterior transpetrosal approach (ATPA), also known as the Kawase approach, is a milestone technique that enables surgical access to the petroclival area (4). After its introduction over two decades ago, many studies have demonstrated the surgical applications and further modifications of this technique (5–8).

On the other hand, with the extreme development of minimally invasive surgery using an endoscope, endoscopic surgery is currently regarded as the optimal strategy for treating several skull base lesions. The endoscopic transorbital approach (ETOA) is an emerging surgical route in the neurosurgical field for accessing the skull base in the anterior and middle cranial fossa (9–11). More recently, access to the petrous apex and posterior fossa through the ETOA has been described in the literature (12, 13).

While accessing the posterior fossa, both the ATPA and ETOA employ the same bony landmarks during petrous apex drilling (13). However, due to their contrasting surgical axes, they are expected to show differences in surgical view, maneuverability, and clinical implications. In this study, we performed a comparative cadaveric analysis of ATPA and ETOA. The objective was to investigate the feasibility of ETOA in accessing the petrous apex and posterior fossa, and to compare the surgical view and maneuverability of each approach, thereby helping surgeons to decide the appropriate surgical approach.

## 2 Methods

Quantitative analysis of four adult cadaveric specimens (eight sides in total) was performed to compare the surgical characteristics of transcranial and endoscopic transorbital petrosectomies. For each cadaver head, ATPA was performed on one side and ETOA on the other side.

All cadaveric specimens were prepared with authorization from the Department of Anatomy and the Institute for Applied Anatomy at the Catholic University of Korea. All studies were conducted in accordance with the tenets of the Declaration of Helsinki.

### 2.1 Surgical approach

#### 2.1.1 Transcranial ATPA

**Figure 1:**
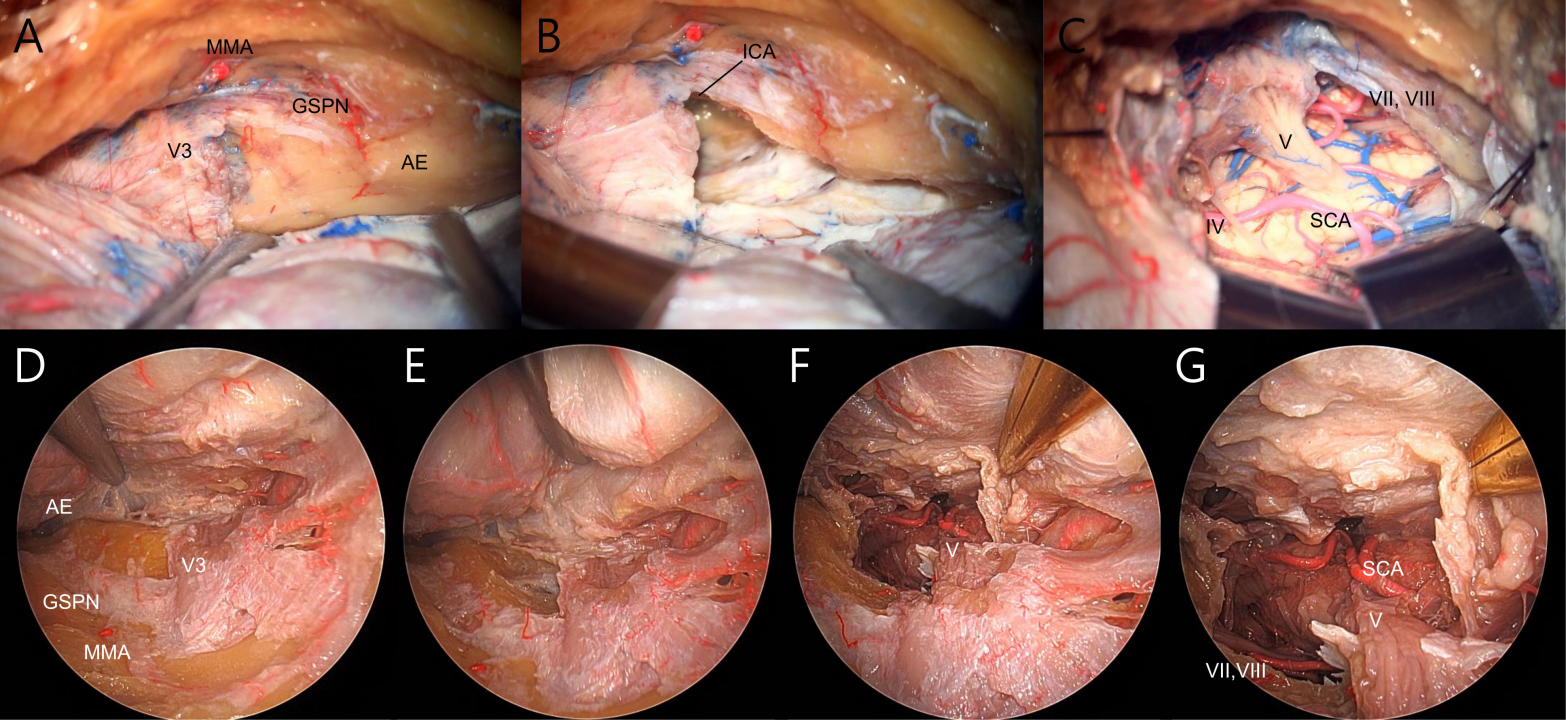
Demonstration of transcranial **(A–C)** and endoscopic transorbital **(D–E)** petrosectomy on the right side. **(A)** The temporal dura was dissected from middle fossa floor and lateral wall of the cavernous sinus, and the landmarks of Kawase rhomboid were delineated. **(B)** The posterior fossa dura was exposured after anterior petrosectomy. **(C)** Exposure of the brainstem after dura and tentorial incision. **(D)** Extradural dissection of temporal dura facilitated complete visualization of the lateral cavernous sinus wall and Kawase rhomboid. **(E)** Kawase rhomboid was drilled and posterior fossa dura was exposed. **(F, G)** Brainstem and posterior fossa were visualized after dura and tentorial incision. MMA, middle meningeal artery; V3, mandibular branch of the trigeminal nerve; GSPN, greater superficial petrosal nerve; AE, arcuate eminence; ICA, internal carotid artery; IV, trochlear nerve; V, trigeminal nerve; VII, facial nerve; VIII, vestibulocochlear nerve; SCA, superior cerebellar artery.

Dissection was performed following a previously described technique (4, 7, 14). A curvelinear incision was made using the landmark of the tragus and midpupillary line. An interfascial dissection of the temporalis fascia was carried out to preserve the frontal branch of the facial nerve. Following the subperiosteal dissection of the temporalis muscle, the posterior root of the zygoma was exposed. A frontotemporal craniotomy with zygomatic osteotomy was performed and the temporalis muscle was retracted inferiorly. The remaining part of the temporal base and sphenoid ridge were drilled down to the middle fossa floor to improve the surgical view. The temporal dura was elevated in posterior-to-anterior and lateral-to-medial directions, and the middle meningeal artery was cut at the level of the foramen spinosum. A sharp incision was made on the lateral wall of the cavernous sinus. By interdural dissection, the dura propria was separated from the membranous dura, covering three divisions of the trigeminal nerves and Gasserian ganglion. Sharp dissection over the greater superficial petrosal nerve (GSPN) was performed and the landmarks of the Kawase rhomboid were delineated (**Figure 1A**) (7). Drilling of the rhomboid area proceeded with medial mobilization of the mandibular division of the trigeminal nerve to maximize petrous removal (**Figure 1B**). The temporal and posterior fossa dura were cut along with division of the superior petrosal sinus. After identifying the trochlear nerve, the tentorium was divided to the tentorial notch, allowing surgical access to the posterior fossa (**Figure 1C**).

#### 2.1.2 ETOA

ETOA was accomplished using a rigid endoscope with a 4 mm diameter, 18 cm length and 0-degree lens (Karl Storz, Tuttlingen, Germany). The superior eyelid approach was performed as previously described (15–17). A linear skin incision was made through the eyelid crease and extended laterally beyond the lateral orbital rim. The eyelid and orbicularis oculi muscles were reflected superior-laterally, and the superolateral orbital rim and fronto-zygomatic sutures were identified. Dissection of the temporalis muscle away from the lateral orbital rim was performed, followed by removal of the lateral orbital rim and the anterior lateral orbital wall. The periorbita was carefully dissected in the subperiorbital plane toward the orbital apex. Throughout the procedure, the orbital contents were gently retracted medially to avoid exceeding a distance of 1 cm from the lateral orbital wall. Under endoscopic visualization, the lateral orbital wall was drilled to expose the extracranial temporalis muscle. Further drilling of the greater and lesser sphenoid wings from the lateral sphenoid ridge was conducted until the temporal pole dura was identified. After identifying the superior and inferior orbital fissures, the sphenoid bone was sufficiently drilled and extended down to the inferior orbital fissure and above the superior orbital fissure. After confirming wide exposure of the frontal base and temporal pole dura, the meningo-orbital band was excised to facilitate interdural dissection by unlocking the lateral wall of the cavernous sinus. The temporal lobe was elevated extradurally, and the continuation of the dissection facilitated the exposure of the entire lateral wall of the cavernous sinus to the trigeminal porus and petrous ridge (**Figure 1D**). The middle meningeal artery was cut and the GSPN was dissected from the dural attachment. This allowed us to drill the Kawase rhomboid to expose the dura of the posterior fossa (**Figure 1E**). Wide exposure of the brainstem was achieved by dividing the tentorium after incision of the temporal and posterior fossa dura (**Figures 1F, G**).

### 2.2 Quantitative measurement

For each specimen, a 0.7 mm thick axial spiral CT scan was obtained before and after dissection. Stereotactic measurements using neuronavigation (Stealth Station; Medtronic Sofamor Danek, Memphis, TN, USA) were performed on each target of interest. The measurements consisted of 3-dimensional positional information with Cartesian coordinates X, Y, and Z. All retrieved data were computed using Microsoft Excel spreadsheet software (Microsoft Office Excel; Microsoft Corp., Redmond, Washington, USA) for further analysis.

#### 2.2.1 Angle of attack (AOA) and surgical depth

The angle of attack was defined as the maximal maneuverability of the instruments on the target of interest in the vertical and horizontal planes. For each approach, the root exit zone (REZ) of the trigeminal and facial nerves was used as the measurement target. During ATPA, the REZ of the abducens nerve was also assessed. For the calculation, the distal end of the instrument was fixed to the target, while the proximal end was moved as far as possible in each plane. Accordingly, the coordinates of the position of the proximal end of the instrument were obtained using stereotaxis.

The depth of the surgical corridor was measured from the bony surface to the target used for the AOA measurement. The bony surfaces used were the level of the outer table of the temporal bone for ATPA and the junction of the upper edge of the zygomatic arch and lateral orbital rim for ETOA.

#### 2.2.2 Area of brainstem exposure

**Figure 2:**
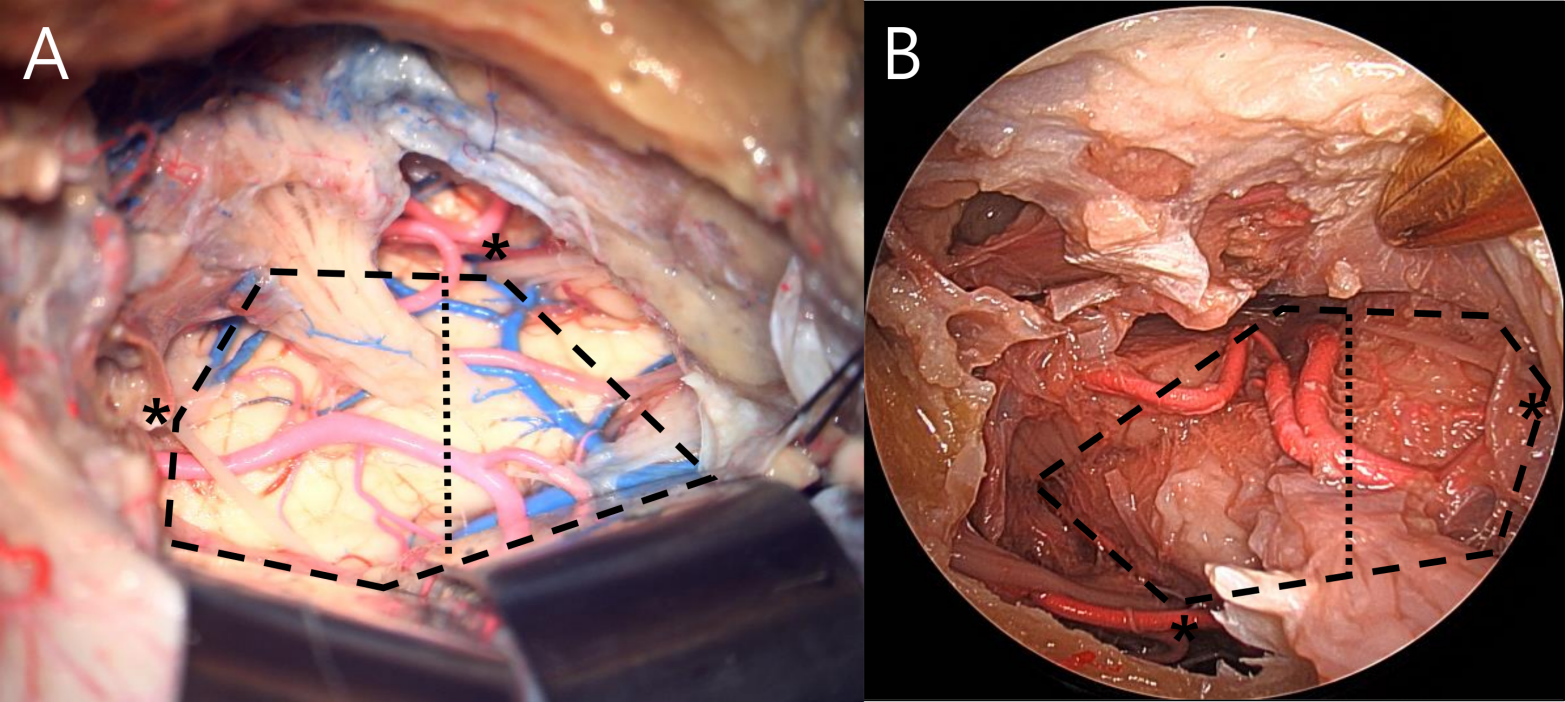
Area of brainstem exposure measured by analyzing hexagonal area in anterior transpetrosal **(A)** and endoscopic transorbital **(B)** approach. Asterisk indicates fixed landmarks. Measured area was divided into anterior and lateral brainstem by the longitudinal line crossing the entry point of trigeminal root (dotted line).

The area of brainstem exposure under visualization by either microscope or 0-degree endoscope was defined as the imaginary hexagonal area connecting six anatomical landmarks after dura and tentorial resection (**Figure 2**). The first two fixed landmarks were assigned to the dural entrance of the trochlear nerve and the REZ of the facial nerve. The other four variable landmarks were allotted to the following points: the most anterosuperior, anteroinferior, and posterosuperior accessible points of the brainstem, and the most posterior accessible point of the trochlear nerve (13). In addition, the exposed brainstem was divided into two areas (anterior and lateral brainstem) based on the longitudinal line crossing the entry point of the trigeminal root, and the area of each was measured.

### 2.3 Statistical analysis

All measurements are presented as mean ± standard deviation (SD). Comparisons between two groups were performed using the Student’s t-test or Mann-Whitney test. A *P*-value of <0.05 indicated statistical significance. All statistical analyses were performed using R version 3.3.3 (http://www.r-project.org/) and SPSS (version 18.0; SPSS Inc., Chicago, IL, USA).

## 3 Results

**Figure 3:**
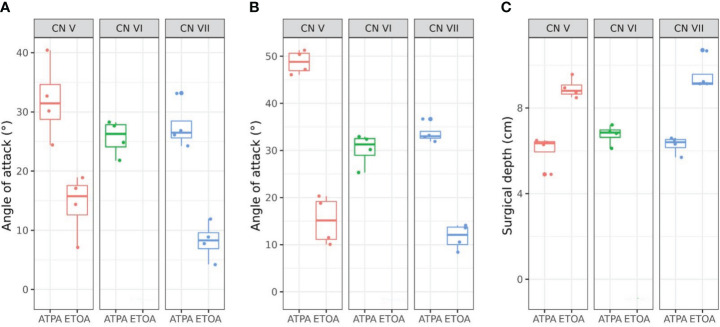
Box plots with scatter plots of the vertical angle of attack (AOA) **(A)**, horizontal AOA **(B)** and surgical depth **(C)** of each approach. CN, cranial nerve; ATPA, anterior transpetrosal approach; ETOA, endoscopic transorbital approach.

The measurements of AOA and surgical depth of the two approaches are shown in **Table 1**, and also presented in **Figure 3**. ATPA showed significantly greater value at the trigeminal REZ in both vertical (31.8 ± 6.7° vs. 14.3 ± 5.3°, p=0.006) and horizontal AOA (48.5 ± 2.9° vs. 15.0 ± 5.2°, p<0.001) than ETOA. AOA at facial REZ was also greater in ATPA than ETOA (vertical, 27.5 ± 3.9° vs. 8.3 ± 3.3°, p<0.001; horizontal, 33.8 ± 2.2° vs. 11.8 ± 2.9°, p<0.001). In addition, ATPA was associated with significantly shorter surgical depth (CN V, 5.8 ± 0.5 cm vs. 9.0 ± 0.8 cm, p<0.001; CN VII, 6.3 ± 0.5 cm vs. 9.5 ± 1.0 cm, p=0.001) than ETOA.

**Table 1:** Angle of attack and surgical depth measured by each approach.

		ATPA	ETOA	p-value	95% CI
Angle of attack (°)					
CN V					
Vertical		31.8 ± 6.7	14.3 ± 5.3	0.006	7.131 – 27.869
Horizontal		48.5 ± 2.9	15.0 ± 5.2	<0.001	25.668 – 41.332
CN VI					
Vertical		25.8 ± 2.9	N/A		
Horizontal		30.0 ± 3.6	N/A		
CN VII					
Vertical		27.5 ± 3.9	8.3 ± 3.3	<0.001	13.022 – 25.478
Horizontal		33.8 ± 2.2	11.8 ± 2.9	<0.001	17.561 – 26.439
Surgical depth (cm)					
CN V		5.8 ± 0.5	9.0 ± 0.8	<0.001	-4.421 – -2.079
CN VI		6.8 ± 0.5	N/A		
CN VII		6.3 ± 0.5	9.5 ± 1.0	0.001	-4.618 – -1.882

ATPA, anterior transpetrosal approach; ETOA, endoscopic transorbital approach; CI, confidence interval; CN, cranial nerve.

**Figure 4:**
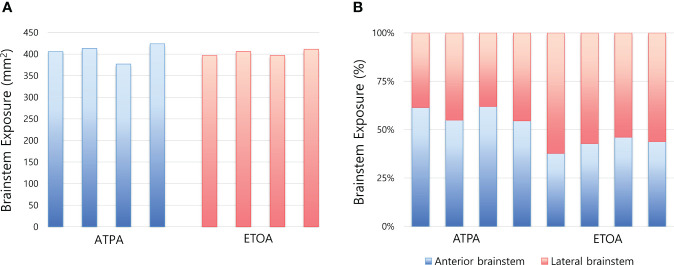
Box plots comparing brainstem exposure between two approaches. **(A)** Area of brainstem exposure of each measurement. **(B)** Difference in the proportion of brainstem exposure between two approaches. ATPA, anterior transpetrosal approach; ETOA, endoscopic transorbital approach.

The results for the area of brainstem exposure are shown in **Table 2**, and the same data were presented in **Figure 4**. The mean areas of exposure did not differ between ATPA and ETOA (404.8 ± 20.0 mm^2^ vs. 402.8 ± 7.0 mm^2^) (**Figure 4A**). However, when the area was divided into the anterior and lateral brainstem, the proportion at which each area was exposed differed between the two approaches (**Figure 4B**). ATPA showed significantly better exposure of the anterior brainstem than ETOA (240.7 ± 9.6 mm^2^ vs. 171.7 ± 15.0 mm^2^, p<0.001), while ETOA demonstrated better lateral brainstem exposure (174.2 ± 29.1 mm^2^ vs. 231.1 ± 13.6 mm^2^, p=0.022). In ATPA, the mean area of exposure was 404.8 ± 20.0 mm^2^, of which, the area of anterior brainstem was 240.7 ± 9.6 mm^2^, accounting for 59.5%. Contrastively, ETOA mainly exposed the lateral brainstem (231.1 ± 13.6 mm^2^), and the area medial to trigeminal root only covered 42.6% of the total area.

**Table 2:** Area of brainstem exposure measured by each approach.

	ATPA	ETOA	p-value	95% CI
Brainstem exposure (mm^2^)	404.8 ± 20.0	402.8 ± 7.0	0.851	-23.908 – 28.064
Anterior brainstem	240.7 ± 9.6	171.7 ± 15.0	<0.001	47.164 – 90.786
Lateral brainstem	174.2 ± 29.1	231.1 ± 13.6	0.022	-100.374 – -13.376

ATPA, anterior transpetrosal approach; ETOA, endoscopic transorbital approach; CI, confidence interval.

## 4 Discussion

This study aimed to evaluate the viability of the lateral orbital corridor as an alternative option to access the petrous apex and posterior fossa, as well as to compare the surgical nuances of ETOA with ATPA. The results of the current study verified that anterior petrosectomy through the ETOA provides an adequate corridor to the petroclival area and anterolateral brainstem, along with acceptable maneuverability and surgical exposure. However, because ATPA and ETOA exert different surgical trajectories, they show dissimilar surgical exposure and maneuverability. Our morphometric analysis focused on the differences in the surgical view of each access according to the surgical trajectory, as well as the quantitative measurements of each approach.

The ATPA is traditionally an extradural approach that is beneficial in approaching the petroclival area without extensive brain retraction and with little damage to vital neurovascular structures while preserving hearing (3, 18). Nevertheless, ATPA may be subject to a number of complications because the internal carotid artery and nearby neuro-otological structures are potentially at risk from extensive bone drilling (19). Other possible complications include injury to the GSPN and temporal lobe contusions (20). However, lumbar drain placement, the use of dynamic retraction avoiding the use of spatula and the new inter-dural technique permit to reduce this risk (21).

Recently, increased focus has been placed on ETOA to the skull base in accordance with an effort to circumvent these shortcomings of the transcranial approach (22–24). Only minimal displacement of the orbit is required, and the surgical pathway does not involve any brain retraction or conflict with major neurovascular structures (25, 26). Various type of ETOA have been previously described, including the precaruncular, preseptal lower eyelid, lateral retrocanthal, and superior eyelid crease approaches (27). In addition, the ETOA has undergone several modifications with the addition of lateral orbital rim osteotomy, providing tremendous surgical benefits, such as a wider range of movement and lesser orbital retraction, despite some morbidities (13, 28). In the current study, we performed a superior eyelid crease incision, which is the most commonly adopted technique in the neurosurgical field. We also removed the lateral orbital rim to compare and analyze the maximum maneuverability and surgical exposure of each approach.

The mean AOA offered by the ETOA was significantly lower than that offered by the ATPA. This was recognized to be a consequence of not only the restricted space of the corridor but also the significantly longer surgical depth from the eyelid to the surgical target in the posterior fossa (13). At each target, the mean surgical depth of the ETOA was significantly longer than that of the ATPA by more than 3 cm. It is also considered to be resulted in an angular relationship between the surgical axis and petrous ridge. In contrast to ATPA, which allows lateral access to the posterior fossa by a perpendicular approach to the petrous ridge, ETOA has an antero-posterior corridor, reducing maneuverability through the area of Kawase rhomboid.

Both ATPA and ETOA resulted in equivalent exposure of the anterolateral brainstem, which is consistent with the findings of previous studies (13). However, when analyzing the area separated by a longitudinal line crossing the trigeminal root, ATPA showed significantly greater exposure of the anterior brainstem than ETOA. In contrast, ETOA facilitates a greater mean area of the lateral brainstem than ATPA, while access to the anterior brainstem medial to the trigeminal nerve is limited. Although the petroclival area can be accessed through both the ATPA and ETOA, it is advocated that the ATPA provides a descending trajectory, whereas the ETOA gives parallel angle toward the posterior cranial fossa (29). Since each approach employs a contrasting surgical corridor, there was an apparent difference in the surgical view provided at the same area of the brainstem. The parallel angle of view offered by ETOA, which provides surgical trajectory along the lateral border of the trigeminal nerve, allows a limited view of the anterior brainstem, especially medial-inferior to the trigeminal root. Consequently, it is not possible to locate the REZ of the abducens nerve, which is identifiable in the ATPA. Since abducens nerve is involved in most lesions located in the anterolateral brainstem, missing the exposure of the its origin is regarded to be a significant limitation of ETOA. The facial nerve could be identified after exposure in both approaches, but it appeared to emerge toward the operator in ATPA. On the other hand, ETOA has the advantage of allowing a view the entire path of the facial nerve.

The results of measurements confirmed that the AOA and surgical depth were superior in ATPA than in ETOA. Although ETOA provided comparable brainstem exposure by providing a panoramic view inherent to the endoscope, the available surgical maneuverability was reduced, making bimanual surgical maneuvers more difficult. Moreover, the antero-posterior view of the ETOA complicates upward retraction of the temporal base dura during the drilling of the petrous apex through a limited space allowed by the lateral orbital corridor. In addition, dissection of the GSPN along with temporal dura in a proximal-to-distal direction from the anterior endoscopic view provides a certain degree of difficulty. Nevertheless, it was shown to be preferable to ATPA regarding the straight route to the brainstem, lateral to the trigeminal nerve. Therefore, despite several disadvantages, ETOA may be considered a valid surgical option to access the brainstem for selected lesions primarily involving the lateral to trigeminal nerve or in cases of anterior-to-posteriorly projected lesions in the anterolateral cerebellopontine angle in patients who require minimal invasiveness or have cosmetic concerns.

The strength of current study lies that, in contrast to earlier studies, it verified the difference in surgical view caused by the discrepancy in the surgical corridor between two approaches rather than merely comparing the exposed area of the brainstem.

This study has certain limitations inherent to anatomical studies. Owing to the limited number of available specimens, our results cannot be generalized. Moreover, since each approach was performed on either side of the cadaver head, any asymmetry could lead to inaccurate results. In addition, given that cadaveric models cannot fully replicate the surgical setting, tissue characteristics, presence of cerebrospinal fluid, and anatomical distortions that may occur due to pathologic processes, the measurements should be interpreted cautiously.

## 5 Conclusions

Because the surgical trajectory and exposure of ATPA and ETOA are contrasting, the selection of the surgical approach must be precisely discussed. The current study confirmed that ETOA could be a valid minimally invasive technique that provides a direct ventral route to the brainstem. Compared with ATPA, ETOA showed less surgical maneuverability, AOA and longer surgical depth; however, it presented comparable brainstem exposure with better exposure of the lateral brainstem. Therefore, ETOA should be cautiously attempted in appropriate cases for access to the petroclival area and anterolateral brainstem.

## Data availability statement

The original contributions presented in the study are included in the article/supplementary material. Further inquiries can be directed to the corresponding author.

## Author contributions

YN contributed the conceptualization, supervision, and project administration. SK, MN contributed to the methodology, formal analysis, data curation, and writing the original draft. K-SC, HB contributed to the validation, writing, review, and editing. All authors contributed to the article and approved the submitted version.
